# Non-Intrusive System for Honeybee Recognition Based on Audio Signals and Maximum Likelihood Classification by Autoencoder

**DOI:** 10.3390/s24165389

**Published:** 2024-08-21

**Authors:** Urszula Libal, Pawel Biernacki

**Affiliations:** Department of Acoustics, Multimedia and Signal Processing, Wroclaw University of Science and Technology, 50-370 Wroclaw, Poland

**Keywords:** beehive monitoring, smart beehives, anomaly detection, power spectral density, signal processing, artificial intelligence, autoencoder neural network

## Abstract

Artificial intelligence and Internet of Things are playing an increasingly important role in monitoring beehives. In this paper, we propose a method for automatic recognition of honeybee type by analyzing the sound generated by worker bees and drone bees during their flight close to an entrance to a beehive. We conducted a wide comparative study to determine the most effective preprocessing of audio signals for the detection problem. We compared the results for several different methods for signal representation in the frequency domain, including mel-frequency cepstral coefficients (MFCCs), gammatone cepstral coefficients (GTCCs), the multiple signal classification method (MUSIC) and parametric estimation of power spectral density (PSD) by the Burg algorithm. The coefficients serve as inputs for an autoencoder neural network to discriminate drone bees from worker bees. The classification is based on the reconstruction error of the signal representations produced by the autoencoder. We propose a novel approach to class separation by the autoencoder neural network with various thresholds between decision areas, including the maximum likelihood threshold for the reconstruction error. By classifying real-life signals, we demonstrated that it is possible to differentiate drone bees and worker bees based solely on audio signals. The attained level of detection accuracy enables the creation of an efficient automatic system for beekeepers.

## 1. Introduction

Swarming is a natural phenomenon that occurs when an *Apis mellifera* honeybee colony reproduces and splits into multiple colonies. Swarming typically occurs during the late sping and early summer months [1,2,3,4]. The traditional beekeeping approach requires systematic beehive monitoring by beekeepers who observe the states of the colonies and take necessary actions to prevent swarming. Nowadays, new automated approaches to beehive monitoring are being developed that utilize machine learning techniques to build an intelligent monitoring system. Non-invasive automated detection of Varroa mites [5] and early detection of swarming [6,7] are among the topics most actively studied in beekeeping. Swarming in honeybees can be expected after a beekeeper has observed new queen cells inside a hive. The other indicators leading to swarming are a higher foraging activity, a rapid growth of the colony, and drone bee appearance outside of a hive.

Thus, the detection of swarming without interfering with the hive can be based on the detection of drone bees’ activity around the hive during the mating season. Each summer, thousands of male bees, the drones, travel to drone congregation areas [3,7,8,9,10], attempting to mate with virgin queens from neighboring hives, which correlates with their higher detectability around entrances to the hives. The share of drones in a bee family during the mating season can increase up to 15% of the bee colony—Ref. [2] stated that the honeybee colony rather precisely limits the drone comb to some 15% of the total comb within its hive. There have been studies on honeybees’ activities, including automatic monitoring systems counting forager bees entering and leaving the hives, proposed by [11], or drone activity monitoring in [12]. Therefore, based on the facts presented, an early-stage swarming alarm system for beekeepers can be constructed by analyzing bee sounds around a hive and identifying drones.

Honeybees use sound as a means of communication, both within the hive and with other bees outside the hive. The sound produced by bees is a form of vibration created by the rapid beating of their wings and is used to convey information about the location of food, the presence of danger, and other important information. One of the most well-known sounds produced by bees is the buzzing sound that is heard when they are in flight. This sound is created by the rapid beating of their wings, which can occur at a rate of approximately 260 Hz [2], i.e., 260 wing beats per second.

**Figure 1:**
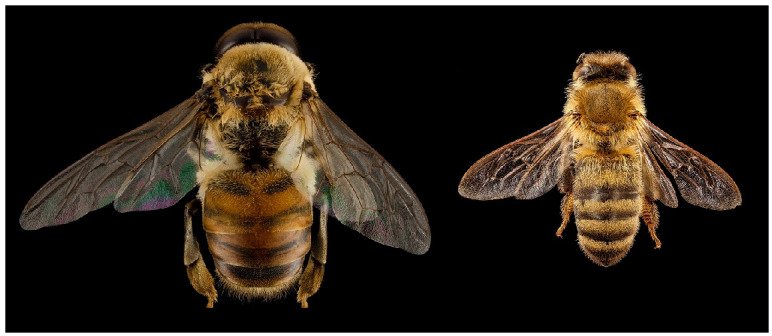
Honeybees *Apis mellifera* L.: male drone (**left**) and female worker bee (**right**). Photography: Beltsville Agriculture Research Center, public domain.

To distinguish between worker bees and drones, based on the sounds they make, one can use the fact that the drones are generally bigger and have longer wings (see Figure 1). The results shown for bumblebees by [13] indicate that the body shape or the wing size of a pollinator can be correlated with the fundamental frequency it produces. Additionally, by examining the frequency spectrum or mel-frequency cepstral coefficients (MFCCs) [14], it is possible to automatically recognize pollinating bees.

### 1.1. Internet of Things Solutions in Beehive Monitoring

In recent years, there has been a significant shift from manual beehive monitoring by beekeepers to the application of Internet of Things (IoT) technologies. The primary objectives of these advancements are to enable remote observation of bee colony conditions and to enhance the safety and health monitoring of bees. Additionally, the implementation of more sophisticated methods in beekeeping allows for the collection of large datasets, which facilitate research into the still poorly understood behaviors of honeybees.

Numerous commercial IoT systems and devices have been developed to enhance beekeeping practices, as detailed in [15,16]. These include **Beebot**, which measures hive weight, humidity, and internal temperature; **Easy Bee Counter**, which utilizes 48 infrared (IR) sensors to count bees entering and exiting the hive; and the **Bee-Shop Camera Kit**, designed to capture photos and video recordings that can be stored on an SD card or transmitted to a beekeeper’s mobile device via the 3G/4G LTE network. Additionally, **EyeSon Hives** employs an image detection algorithm to analyze swarm flight direction, offering real-time video monitoring through 3G/4G LTE connectivity. Other notable devices include **Zygi**, which measures temperature, humidity, and weight; **Hive-Tech**, which detects swarming activity using IR and reflectance sensors to monitor real-time crowd conditions; and **HiveMind**, which tracks bee activity using sound and IR sensors.

Another noteworthy example is the self-powered **SBMaCS system** [17], which incorporates temperature, humidity, weight, motion, and flame sensors. A system described in [18] uses a thermal camera to count bees, employing four popular classifiers: k-nearest neighbors, neural networks, random forest, and support vector machine. Additionally, bee classification using an autoencoder neural network trained on mel-frequency cepstral coefficients (MFCCs) as representations of audio samples was proposed in our previous works [19,20,21].

The **AppMAIS project** [22] was developed to better understand and prevent colony collapse disorder, which leads to the rapid loss of adult worker bees and sudden colony mortality. AppMAIS is based on the open-source IoT platform Thingsboard, which generates alerts upon detecting specific colony states. As highlighted in the survey in [7], energy consumption and non-intrusiveness are crucial aspects of such systems.

IoT technology is closely related to radio-frequency identification (RFID) technology. As noted in [23], RFID tags can be used for continuous monitoring of individual bees within a colony, offering a less time-consuming alternative to video surveillance. However, this approach involves attaching RFID tags to the backs of individual bees, which is challenging due to their small size. Considering that an average colony consists of 20,000 to 80,000 bees, a large number of RFID tags would be required, and the data obtained would only provide information about a bee entering or leaving a specific area defined by the location and parameters of an RFID reader.

### 1.2. Audio Analysis Methods

Mel-frequency cepstral coefficients (MFCCs) are the most common set of features used in numerous studies [24,25,26,27] that exploit a machine learning framework based on audio recordings. In the mel-frequency cepstral method of obtaining a power spectrum representation of a signal, the frequency bands are equally spaced on the mel scale. Many studies analyzed MFCCs to extract information for bee detection, queen absence and swarming detection, and bee species identification, as well as environmental effects, with the three first coefficients showing the highest discrimination potential.

Despite the popularity of MFCCs, methods based on power spectral density (PSD) have also been introduced to honeybee sound analysis. In a study on drone audio detection [19], parametric and non-parametric methods of PSD estimation were compared for various frequency bandwidths, ranging from 100 Hz to 3000 Hz. In [28], the short-time Fourier transform (STFT) calculated with filter banks and the overlapping method was used to detect swarming events. The STFT was calculated using 128, 256, 512, and 1024 bins to investigate the impact of window width.

### 1.3. Machine Learning Algorithms

A typical machine learning framework encompasses signal measurement, preprocessing, feature extraction, and lastly, classification. In the area of acoustic analysis of bee sounds, many different classifiers have been explored, with the most common classifier—a support vector machine (SVM) [29,30,31]—as en example. The SVM is a kernel-based method that projects data into higher dimensions in which a hyperplane can effectively separate classes.

But recently, neural network-based deep learning [32] methods have been willingly introduced. In [33,34], the use of so-called long short-term memory (LSTM) recurrent neural networks (RNNs) for queen bee presence detection is proposed. A comparison between an LSTM, a multilayer perceptron (MLP) neural network, and logistic regression was made, and it showed the power of the LSTM for the task at hand. Recently, convolutional neural networks (CNNs) [31,35,36] have gained popularity, especially within computer vision tasks. To make them directly applicable to bee acoustics analysis, researchers have relied on image-like inputs, such as spectrograms, mel-scaled spectrograms, or other two-dimensional time–frequency representations of the audio signals.

### 1.4. Data Acquisition and the Proposed Processing

This paper presents results based on a selected set of audio recordings acquired by our proposed beehive monitoring system. It is designed to identify and predict certain events and states of the beehive that are of interest to the beekeeper.

Data were collected twice a day during the highest outdoor activity of worker bees foraging pollen and nectar (around from 8 am to 10 pm and from 3 pm to 5 pm). Bees generally avoid flying during rain or windy weather conditions for safety reasons. To prevent abnormal bee behavior, rain damage to equipment, and wind interference with recordings, all recordings were collected on sunny, almost windless days in June (early summer in the Northern Hemisphere). A directional microphone, facing the entrance to the beehive, was mounted at the edge of its roof.

All recordings were sampled at a frequency of 44,100 Hz and stored in an uncompressed 16-bit WAV format. For signal processing purposes, the recordings were segmented into 1 s long intervals. For the purposes of digital signal processing, the recordings were divided into samples of 1 s in length. The dataset utilized in the off-line simulation comprises 3400 flight sound signals of worker bees and about 1700 sound signals of flying drone bees. Our database [37] is available online with open access.

The ground truth was verified using a video camera standing at a tripod aiming at the front of the beehive. Video footage was used to determine which segments of the audio recordings contained unambiguous representations of each class: worker bees or drone bees. Only audio segments that could be clearly classified were included in our database. Due to the manual verification process, the dataset is relatively small. The clear separation between classes contributed to high classification accuracy across all methods. However, we anticipate lower performance in real-world applications for this reason.

**Figure 2:**
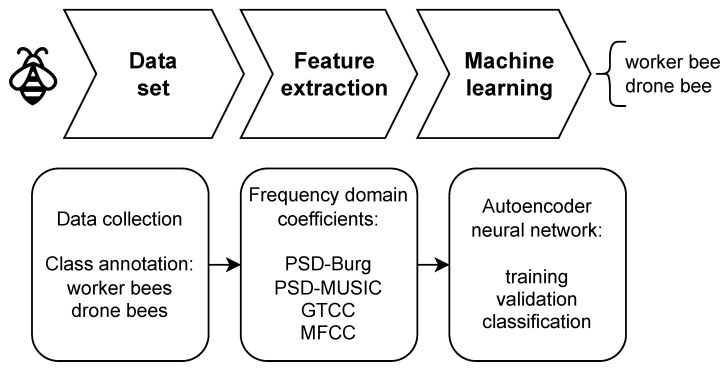
Signal processing diagram.

The overall drone bee detection process is split into two main parts: feature extraction by signal preprocessing and classification with the use of autoencoder neural network reconstruction error. The signal processing diagram, including the detection of bees using the autoencoder neural network, is illustrated in Figure 2.

For feature extraction, we have used two spectral and two cepstral representations of audio signals, obtained by the following algorithms:

- **Burg algorithm**—a parametric power spectral estimation,
- **MUSIC**—a pseudospectrum estimation,
- **MFCCs**—mel-frequency cepstral coefficients, and
- **GTCCs**—gammatone cepstral coefficients.

Our paper is organized into six sections. We start with introducing the drone bee detection problem and the characteristics of the sounds generated by the honeybees. We investigate the methods published in related works in Section 1.2 and Section 1.3 of the paper. In Section 2, we describe the preprocessing methods to be compared, including feature extraction techniques based on power spectrum density estimation in Section 2.1 and cepstral signal analysis in Section 2.2. The specific structure of autoencoder neural network and its anomaly detection potential is explained in Section 2.3. A bayesian approach to classification exploiting autoencoder neural network with maximum likelihood threshold is proposed in Section 3. In Section 4, we present the drone bees detection results. The discussion of the obtained results is in Section 5 and the summary of the paper is placed at the end in Section 6.

### 1.5. The Main Contributions

The manuscript presents a novel method for automatic recognition of honeybee types: worker bees and drone bees, by analyzing audio signals generated by flying bees near a beehive entrance. The main contributions include the following:

- **Audio Signal Processing for Bee Classification**: This study explores various methods for audio signal representation in the frequency domain, including mel-frequency cepstral coefficients (MFCCs), gammatone cepstral coefficients (GTCCs), multiple signal classification (MUSIC), and Burg’s method for parametric estimation of power spectral density (PSD).
- **Use of Autoencoder Neural Network**: The proposed system uses an autoencoder neural network for classifying bees based on the reconstruction error of signal representations. The autoencoder, typically used for generative reconstruction, is repurposed to distinguish drone bees (anomalous signals) from worker bees (normal signals) by leveraging differences in reconstruction error.
- **Novel Thresholding Approach**: The paper introduces a new method for class separation using various thresholds, including a maximum likelihood threshold (T*) to optimize the classification accuracy, achieving near-perfect accuracy in most cases.
- **Empirical Bayes Classifier**: The method effectively acts as an empirical version of the Bayes classifier, minimizing misclassification probability based on reconstruction error histograms.
- **Practical Implications**: The results, obtained under ideal low-noise conditions, suggest that the system could be effectively deployed in real-world beehive monitoring, with plans for integration with data acquisition and communication modules to transmit alerts and status updates to beekeepers.

The research demonstrates the feasibility of differentiating between drone and worker bees using audio signals alone, paving the way for practical applications in beekeeping, such as swarming prevention, with potential enhancements through additional sensors and data transmission via GSM.

## 2. Materials and Methods

### 2.1. Spectral Coefficients

The power spectral density (PSD) function is commonly used in signal processing, including audio signal processing, to analyze the frequency content of a signal. The PSD provides information about how the power of a signal is distributed across different frequency components. Audio signals are typically complex and can contain a wide range of frequencies. The PSD allows us to analyze the distribution of power across different frequency bands. This information is essential for understanding the characteristics of the sound, such as pitch, timbre, and other frequency-related attributes.

The PSD analysis is often used to extract relevant features from audio signals. These features can be used for various applications, including audio classification, speech recognition, and music analysis. For example, different genres of music may exhibit different patterns in the frequency domain.

We decided to use the power spectrum density (PSD) coefficients as features in the learning phase of the neural network in the detection process.

Power spectral density estimation techniques can be divided into parametric and non-parametric methods. The non-parametric methods estimate PSD explicitly from signal samples, without any assumptions about particular process structure. The parametric approaches assume that the signal can be described as the stationary process (MA—moving average; AR—autoregressive; ARMA—autoregressive moving average) of order *m*. Power spectral density is then calculated using the estimated model parameters. This paper presents PSD estimation with the parametric approach by the Burg method and with eigenvector decomposition of an autocorrelation matrix for the MUSIC method.

#### 2.1.1. Burg Algorithm

The Burg algorithm [38,39] assumes that a signal can be described as an autoregressive (AR) process of order *m*:

(1) $$\hat{x}=-\sum_{k=1}^{m}a_{m}\left(k\right)x\left(n-k\right).$$

The Burg algorithm solves the ordinary least squares problem. AR parameters $a_{m}$ are estimated by minimizing the prediction forward and backward errors, which are referred to as the errors between the actual value of the signal and the corresponding estimators in forward and backward directions:

(2) $$PSD_{BURG}\left(f\right)=\frac{E_{m}}{|1+\sum_{k=1}^{m}a_{m}\left(k\right)e^{-j2\pi fk}{|}^{2}}.$$

#### 2.1.2. MUSIC

**Figure 3:**
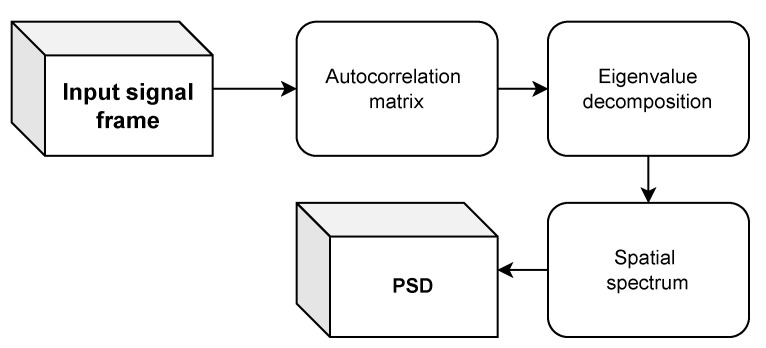
Diagram of PSD estimation by MUSIC.

The MUSIC (multiple signal classification) algorithm calculates the pseudospectrum based on a signal or correlation matrix using Schmidt’s eigenspace analysis technique [40]. This method involves analyzing the eigenspace of the correlation matrix of the signal to determine its frequency characteristics, as presented in Figure 3.

The algorithm is well suited for signals composed of the combination of sinusoids with added white Gaussian noise. Its appeal lies in the following recognized benefits:

- Ability to manage multiple concurrent sound sources. In our case, it is hard to record only one bee or drone. In most cases, we are able to obtain sounds from many species at the same time.
- Precise measurements. Our earlier research [19] has shown that the key to correct recognition lies in detailed spectrum analysis up to a few kilohertz.
- Excellent spatial resolution. In many observations, worker bees and drones are moving, which means in other words that the signal source is changing its position.

### 2.2. Cepstral Coefficients

#### 2.2.1. MFCC

Mel-frequency cepstral coefficients (MFCCs) are widely used in signal processing and speech analysis because of their effectiveness in capturing essential features of the audio signal, especially in the context of human auditory perception. The process involves representing the energy distribution across different frequency bands, emphasizing important spectral characteristics while discarding less relevant information. This feature extraction process is crucial for signal recognition tasks, as it helps highlight the discriminative aspects of the signal.

The MFCC calculation is mainly based on applying mel-scale filter banks to absolute squared value of the fast Fourier transform (FFT) of a windowed signal frame

(3) $$Y\left(m\right)=\sum_{k=0}^{N-1}W_{m}\left(k\right)*{\left|X\left(k\right)\right|}^{2},$$

where X(k) is the FFT of a signal frame (each frame is a vector of *N* samples in time domain), *k* is the FFT bin number, $W_{m}\left(k\right)$ is the *m*th mel-scale filter for m=1,…,M, and *M* is a chosen number of mel-scale filter banks. The formula for converting from frequency in hertz to mel scale is

(4) $$mel\left(f\right)=1127\cdot ln\left(1+\frac{f}{700}\right),$$

where $f=\frac{m\cdot f_{s}}{N}$ is the frequency and $f_{s}$ is the sampling rate.

The last step is an application of discrete cosine transform (DCT) to the obtained output, which gives a set of cepstral coefficients:

(5) $$c_{i}=\sqrt{\frac{2}{M}}\sum_{m=1}^{M}log_{10}\left(Y(m)\right)*cos\left(\left(2m-1\right)\cdot \frac{i\pi }{2M}\right),$$

where *i* is the MFCC coefficient index.

**Figure 4:**
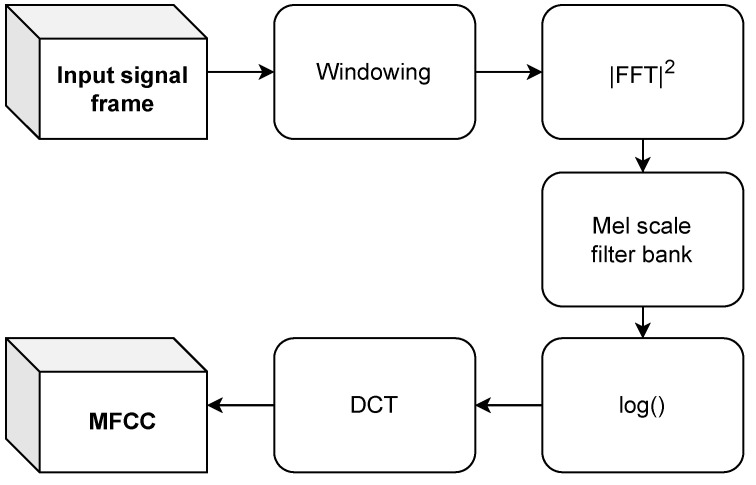
The MFCC extraction diagram.

The block diagram of the MFCC extraction is shown in Figure 4.

#### 2.2.2. GTCC

Gammatone cepstral coefficients (GTCCs) are popular features extracted from audio signals for use in recognition problems. The gammatone function models the response of the human auditory filter. The frequency selectivity properties of the cochlea and those measured psychophysically in humans appear to coincide with properly implemented gamma filters. The gammatone filter bank (composed of the frequency responses of several gammatone filters) emphasizes the perceptually meaningful sound signal frequencies.

The process of calculating gammatone cepstral coefficients is analogous to the MFCC extraction scheme. The gammatone filter bank requires setting the total filter bank bandwidth (BW); gammatone filter order *N*; equivalent rectangular bandwidth model: Lyon, Greenwood, or Glasberg and Moore (ERB); and number of filters. The frequency response of the single filter is defined as follows:

(6) $$GT\left(f\right)={\left(1+j\left(f-f_{0}\right)/BW\right)}^{-N},$$

where $f_{0}$ is a center frequency of the filter.

**Figure 5:**
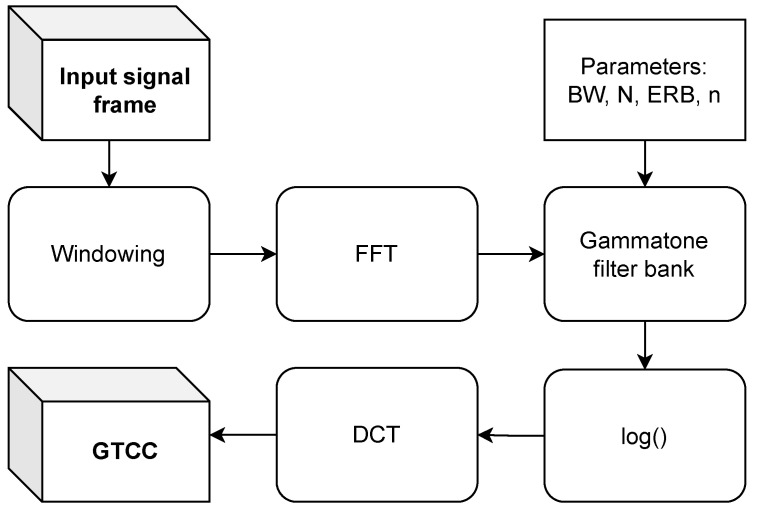
The GTCC extraction diagram.

In Figure 5, we present the block diagram for the GTCC calculation.

### 2.3. Autoencoder Neural Network

For worker bee and drone classification, we propose using an autoencoder. It is a type of generative artificial neural network [41] that can learn the features of the input signals through the training process, during which the model adjusts its parameters to minimize the difference between the input signals and their reconstructions. As the autoencoder minimizes the reconstruction error, it learns to map the input signals to a lower-dimensional representation that captures the essential features of the data. Its weights are adjusted to extract meaningful patterns and structures from the input signals.

The primary goal of autoencoder neural network is reconstruction of signals based on encoded representation. It is mainly used for dimensionality reduction. The previous tests [20,21] have shown that autoencoder neural networks can be modified for the purpose of anomaly detection, in particular for drone bee detection. In Section 3.1, we investigate the further alterations of thresholding of autoencoder reconstruction error, including maximum likelihood threshold.

**Figure 6:**
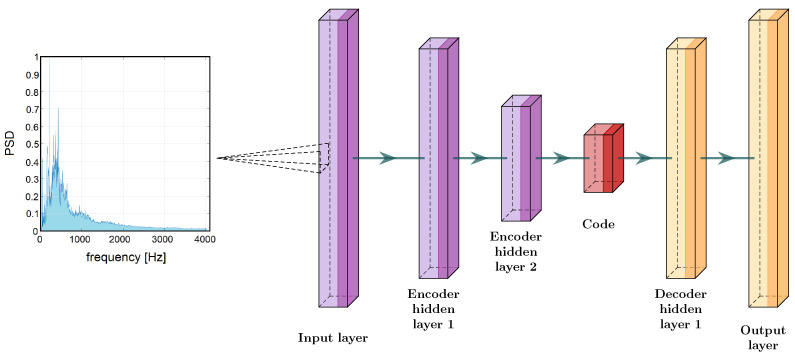
General structure of autoencoder neural network.

The general structure of this network is shown in Figure 6.

For the preprocessed audio recordings using spectral and cepstral methods, we performed a series of numerical experiments, using the neural network of autoencoder type for honeybee classification. The audio signals obtained by the spectral methods (Burg and MUSIC algorithms) were represented by feature vectors counting 512 power spectral density coefficients, and this was the size of the input layer for those cases in the neural network. On the other hand, the cepstral methods have given a lower number of 120 coefficients, which is equal to the number of filter banks in the calculation of the gammatone- and the mel-frequency cepstral coefficients. And for cepstral preprocessing, the input layer size of the neural network was equal to that number. The honeybee classification was tested with four main autoencoder neural network structures: with 1, 2, 3, and 4 hidden layers in the encoder. The size of each layer varied depending on the size of the input layer, but the code layer of the lower size was always described by 8 neurons. In all tests, we applied the most standard setting for anomaly detection by autoencoder, with the decoder having one layer less than the encoder. Except for the output layer that had a sigmoid activation function, all the rest of the layers used ReLu activation functions.

The anomaly detection performed with the use of an autoencoder neural network has the three following main steps:

1. **Training:** In the first step, the autoencoder neural network is trained on feature vectors extracted from worker bee audio recordings only.
2. **Testing:** In the second step, the trained autoencoder is used for reconstruction of training set data, containing both worker bee and drone bee audio recording representations. The reconstruction error is calculated for all signals from the training set.
3. **Classification:** The last step is the classification based on the value of the reconstruction error. The anomaly in the form of drone bee sounds should have a higher reconstruction error.

The classification result depends on the choice of the reconstruction error threshold, dividing the decision areas for the two classes, which is discussed in the next Section 3.

## 3. Theory

### 3.1. Anomaly Detection Threshold

In anomaly detection tasks using autoencoders, the threshold for reconstruction error is typically set based on statistical analysis of the errors obtained from a dataset representing normal conditions. This threshold is often established by using statistical measures like the mean plus a multiple of the standard deviation. This approach ensures that only reconstruction errors significantly higher than those observed for normal data are classified as anomalies. By setting this threshold, the autoencoder can effectively distinguish between normal variations and deviations that suggest anomalies.

The reconstruction error for the worker bees should be significantly smaller since the autoencoder was trained on the worker bees’ feature vectors only. The reconstruction error $MSE^{test}$ for the drone bee feature vectors, applied as an input of the autoencoder in the testing stage, generally achieved higher values, allowing the detection of drones as anomalies for the most commonly occurring worker bees.

**Figure 7:**
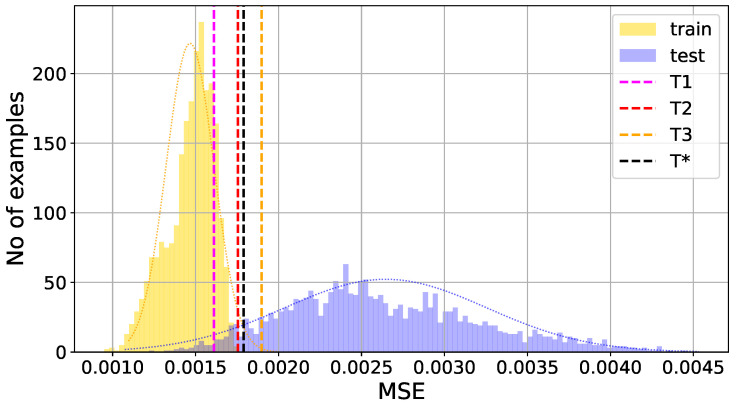
Example of poor class separation: worker bee (training set), drone bee (test set). Histograms of the MSE loss produced by autoencoder neural network with marked threshold values T1, T2, T3, and T*.

**Figure 8:**
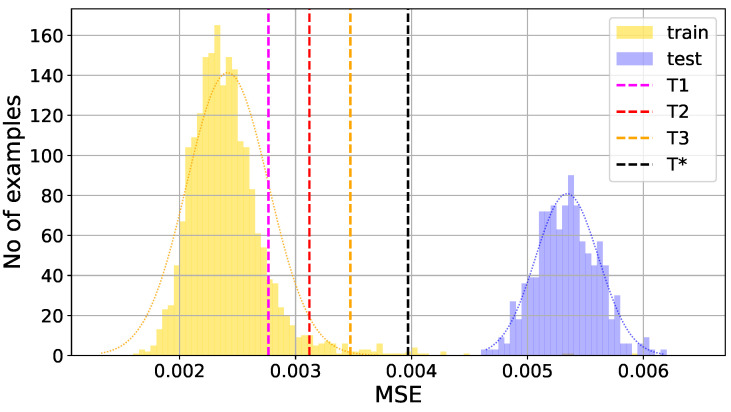
Example of excellent class separation: worker bee (training set), drone bee (test set). Histograms of the MSE loss produced by the autoencoder neural network with marked threshold values T1, T2, T3, and T*.

We performed classification using the following three threshold values:

(7) $$T1=\mathrm{mean}\left(MSE^{train}\right)+\mathrm{std}\left(MSE^{train}\right),$$

(8) $$T2=\mathrm{mean}\left(MSE^{train}\right)+2\cdot \mathrm{std}\left(MSE^{train}\right),$$

(9) $$T3=\mathrm{mean}\left(MSE^{train}\right)+3\cdot \mathrm{std}\left(MSE^{train}\right),$$

where $MSE^{train}$ is reconstruction error for worker bee feature vectors from the training dataset, $mean(MSE^{train})$ is its mean value, and $std(MSE^{train})$ its standard deviation. It should be stressed here that the threshold values are calculated on the basis of results for the training data only, containing the feature vectors for worker bee audio recordings—see Figure 7 and Figure 8. The autoencoder is an unsupervised anomaly detection method, and the testing set containing the coefficient representations of audio signals from the second class, for drone bees, does not take part in the training process. Nevertheless, in this paper, we use the term classification because in the proposed approach, the autoencoder becomes a classifier thanks to the addition of a supplementary step. Autoencoders, though not designed as classifiers, are highly effective in anomaly detection thanks to their generative nature. When an autoencoder is trained solely on data from a specific class, it becomes adept at reconstructing that class while showing a strong sensitivity to deviations. As a result, when data from a different class are encountered, the autoencoder struggles to accurately reconstruct them, flagging these instances as anomalies.

The most popular approach assumes that the threshold value is equal to T1. The threshold defines the decision areas for the classification of worker bees and drone bees, which is executed as a last stage after obtaining the reconstruction error from the autoencoder.

### 3.2. Maximum Likelihood Approach to Classification

We propose a new way of choosing the threshold for autoencoder output, based on the maximum likelihood approach. The optimal classification decision rule is defined as follows:

(10) $$d^{*}\left(x\right)=\max_{c\in \left\{\mathrm{class}\;1,\;\mathrm{class}\;2\right\}}[\;P\left(c\right)\;P\left(x|c\right)\;],$$

where P(c) is the a priori probability and—importantly—*x* is not a feature vector as in a standard classifier, but here, *x* is a reconstruction error, an output of the autoencoder neural network.

The Bayes decision rule $d^{*}$ classifies the output *x* of the autoencoder to a class of higher a posteriori probability, i.e.,

(11) $$d^{*}\left(x\right)=\left\{\begin{array}{cc} \mathrm{class}\;1, & \mathrm{if}\;\;P(1)\;P(x|1)>P(2)\;P(x|2),\\ \mathrm{class}\;2, & \mathrm{if}\;\mathrm{opposite}\;\mathrm{case}. \end{array}\right.$$

The classifier constructed in that way minimizes the expected risk for the zero–one loss function.

**Figure 9:**
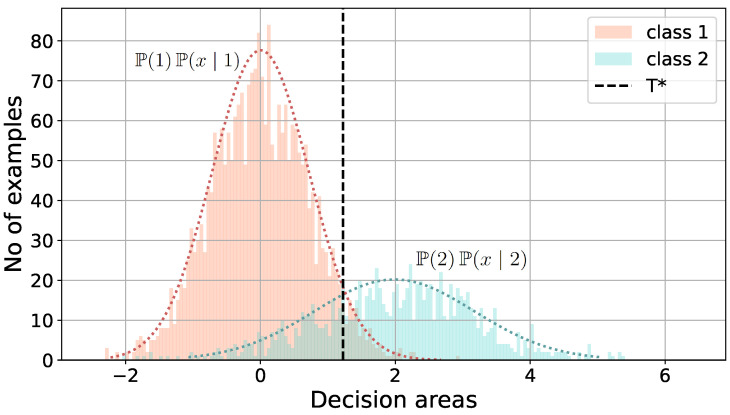
Practical illustration of maximum likelihood approach to the classification with the optimal threshold T* separating the decision areas.

For practical application of that classification rule (11), we based it on probability density function estimation. In our tests, we used Gaussian estimation since the histograms of the obtained MSE from the autoencoder matched the normal distribution quite well. The illustration of determining the optimal threshold T* by fitting Gaussian probability density functions to the data is given in Figure 9.

The proposed threshold values, which separate the decision areas of the classifier, are marked by the dotted vertical lines in Figure 7 and Figure 8, where we present exemplary histograms of the MSE losses from two numerical experiments, returned by the autoencoder neural network for training (worker bees—class 1) and testing (drone bees—class 2) datasets.

The trained autoencoder neural network with the threshold value already calculated is at this point ready to perform class prediction for new input data in a fast way with low computational cost.

### 3.3. Statistical Evaluation

The binary classification by the autoencoder reconstruction error to two classes of worker bees and drone bees, based on their spectral and cepstral representations of feature vectors from the training and test sets, allowed for calculation of the following quality indicators: TP—true positives (number of correctly detected worker bees); TN—true negatives (number of correctly detected drone bees); FP—false positives (number of falsely detected worker bees); and FN—false negatives (number of falsely detected drone bees).

For the verification of the results, based on the four counts listed above, the accuracy of the classifier was calculated. In binary classification, the accuracy is defined as a proportion of correct predictions, being a sum of true positives and true negatives, to the total number of signals from the testing set:

(12) $$Accuracy=\frac{TP+TN}{TP+TN+FP+FN}.$$

In addition to the accuracy, we analyzed also another classifier performance measure, the F1-score. The F1-score is calculated in the following way:

(13) $$F1-score=\frac{2TP}{2TP+FP+FN}.$$

Both accuracy and F1-score can be expressed as percentages, from zero to one hundred. The accuracy is a measure of correct predictions of a classifier, while the F1-score is a harmonic mean of the precision and recall (sensitivity) of the method. The ideal classifier would have an accuracy and F1-score equal to 100%.

## 4. Results

The results presented in this section were obtained for a set of selected 3400 recordings of worker bees and 1700 recordings of drone bees from our database [37] available open access. We extracted feature vectors, as the frequency domain representations of analyzed audio signals, with the use of four methods: two spectral (Burg algorithm and MUSIC) and two cepstral (MFCC and GTCC). As described in the previous section, the experimental classification of worker bees and drones was performed with the use of autoencoder neural networks with 1, 2, 3, and 4 encoder hidden layers, which we will denote by NN1, NN2, NN3, and NN4.

**Figure 10:**
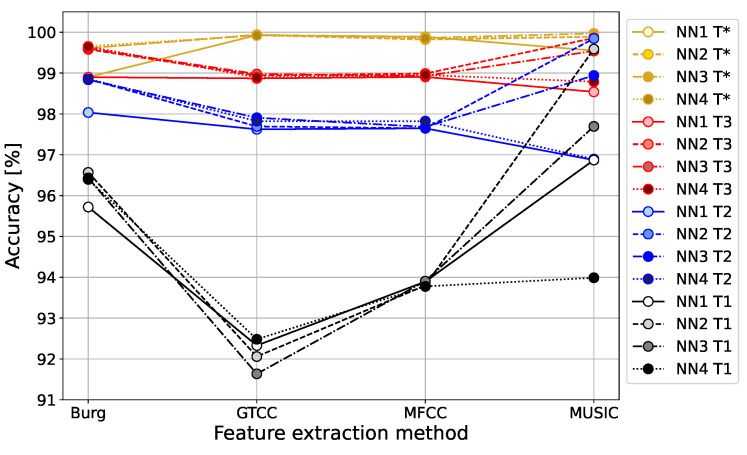
Accuracy of classification of worker bees and drones by autoencoder neural networks with 1, 2, 3, or 4 encoder hidden layers for threshold values: T1, T2, T3.

**Figure 11:**
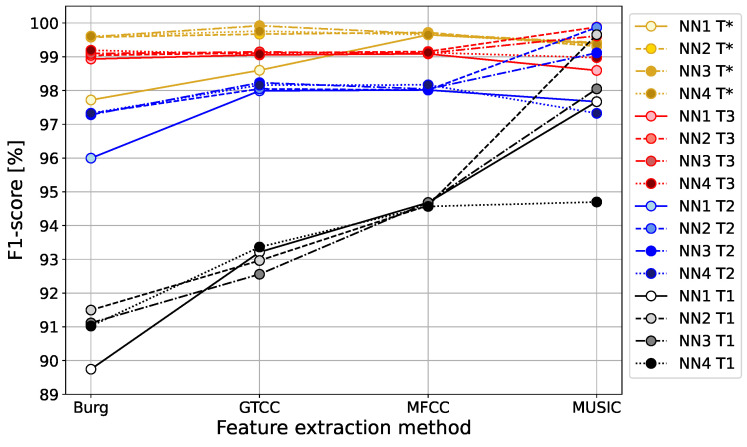
F1-score for classification of worker bees and drones by autoencoder neural networks with 1, 2, 3, or 4 encoder hidden layers for threshold values: T1, T2, T3.

The classification of drone bees and worker bees was carried out with four different autoencoder reconstruction error thresholds T1, T2, T3, and T*, described in Section 3.1 and Section 3.2. The classification accuracy and the F1-score obtained for the three approaches for the thresholds T1, T2, and T3, following the well-known ‘*k*-sigma’ rule dependent on the mean value and the standard deviation of the reconstruction error

(14) $$Tk=\mathrm{mean}\left(MSE^{train}\right)+k\cdot \mathrm{std}\left(MSE^{train}\right),$$

are presented in Figure 10 and Figure 11.

The detailed numerical results for the classification of drone bees and worker bees for all four autoencoder reconstruction error thresholds T1, T2, T3 and the optimal Bayesian threshold T* are shown in Table 1.

## 5. Discussion

Autoencoder neural networks are a specialized form of generative networks designed to copy and compress input data, encoding them in a reduced-dimensional space, and then reconstructing a representation of the original signal (or object) at the network’s output. Although autoencoders are not inherently classifiers, they excel in anomaly detection due to their generative properties. When trained exclusively on data from a single class, the autoencoder becomes highly sensitive to deviations from that class, effectively identifying instances from a second class as anomalies.

In this study, we leveraged this capability by training the autoencoder solely on representations of worker bees. This approach is justified by the fact that worker bees constitute the vast majority of a bee colony, while drone bees typically comprise no more than 15% of the population, and only during the brief swarming period. Consequently, this method simplifies the training process for beekeepers, who can train the network in their specific environments, such as during periods when drone bees are inactive.

**Figure 12:**
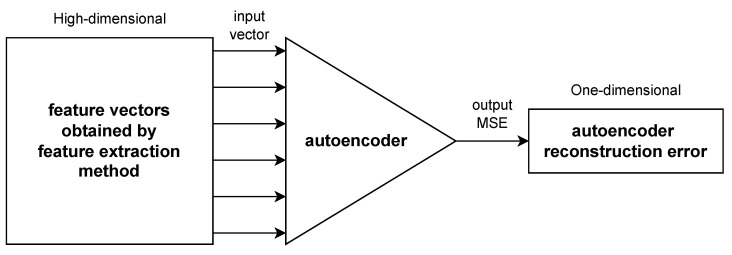
A novel approach to autoencoder neural networks: transformation of high-dimensional classification problem based on feature vectors to a one-dimensional one based on autoencoder reconstruction error.

The proposed usage of the autoencoder neural network as an anomaly detection tool allowed for transformation of the high-dimensional classification problem to a one-dimensional one. The features of honeybee signals were extracted with spectral and cepstral analysis methods, leading to 512-dimensional feature vectors for the Burg and MUSIC methods, and 120-dimensional feature vectors for the GTCC and MFCC extraction methods, due to the frequency band aggregation on the cepstral scale, executed by the mel-scale or the gammatone filter banks. The high-dimensional signal representations were applied in the next step to the autoencoder neural network, whose most common task is generative reconstruction of the input data, but our approach was different. We noticed that for well-separated classes of signals, their representations produced a distinct reconstruction error, low for the worker bees, whose signals were used for training of the autoencoder, and high for drone bees, whose appearance at the entrance to the beehive is quite rare and can be treated as an anomaly. This led us to propose the novel method of classification of honeybees based only on the autoencoder reconstruction error. The standard output of autoencoder neural networks is high-dimensional feature vectors, i.e., generative models mimicking the input data. Our approach based on maximum likelihood (schematically presented in Figure 12), reduced the dimensionality of the problem and protected against the curse of the dimensionality phenomenon, which is a common problem in pattern recognition tasks.

Analyzing the reconstruction error histograms for both classes of signals, worker bees and drone bees (compare Figure 7 and Figure 8), we noticed that for most cases, we had at least good separation of classes. The results for the standard threshold T1 (around 96.28% for Burg method, 92.13% for GTCC, 93.85% for MFCC, and 97.06% for MUSIC) were improved by using the threshold T2 with two standard deviations from the mean value, and later slightly corrected again with threshold T3 with three standard deviations. The reason for that effect is the high separability of the autoencoder MSE in the two classes of signals, and the result is highly data-dependent. It is worth noting that for different datasets with less separate histograms of the output MSE, the thresholds T2 and T3 can potentially lead to worse accuracy than for the standard threshold T1, which is a safe option.

The proposed maximum likelihood threshold T* is a numerically obtained threshold that separates the decision areas of the classifier in the optimal way—see Figure 9. The application of the T* threshold to autoencoder reconstruction error is, in fact, producing an empirical version of the Bayes classifier with the smallest probability of misclassification. In theory, the misclassification probability for the Bayes classifier with full probabilistic information about distributions in classes is the lowest for all classifiers, hence the optimal name. In practice, when the probability distributions in the decision rule (11) must be exchanged with the empirical histograms obtained for the dataset, the misclassification rate will remain the lowest, and at the same time, the complementary accuracy will be the highest. The accuracy results shown in Table 1 confirm the theoretical property of the Bayes classifier for our data. The accuracy for the optimal threshold T* was greater than 99.6% in most cases, except for two: for the Burg and MUSIC preprocessing methods and the neural network structure NN1 with only one hidden layer. The high-dimensional signal representations in the form of 512-dimensional vectors, encoded by only one hidden layer of the neural network, were not as sufficient as encoders with 2, 3, and 4 hidden layers. The optimal threshold T* gives accuracy close to 100%, which means that the classes of worker bees and drone bees, represented by feature vectors obtained by the described methods, are highly separable.

### Prototype of LTE Module

**Figure 13:**
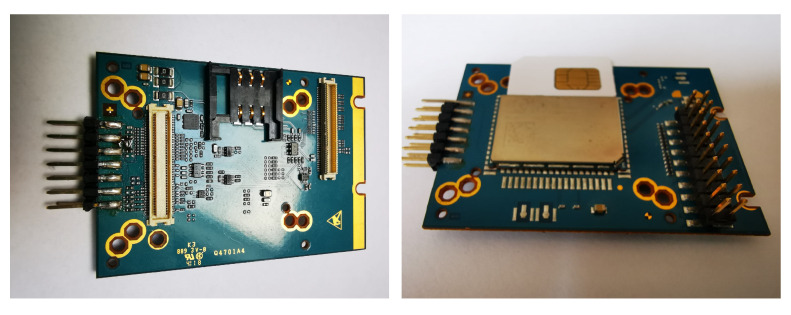
Our evaluation board prototype with LTE module and SIM card slot, designed for data acquisition, signal processing, and wireless communication with a beehive.

The presented signal classification methods will be embedded into an IoT system based on a cellular LTE module. The ultimate goal is to build a functional beehive monitoring system with wireless communication. The device located near the beehive would not only record the audio signals but would also perform the signal processing and send the reports to a beekeeper. Currently, we are working on a prototype of an evaluation board with LTE module. The photo of the board is shown in Figure 13.

The good theoretical results give hope for a practical realization of the proposed system with decent level of accuracy. It should be clearly noted that the results shown in this paper were obtained for a dataset with selected clean signals, recorded in low-noise conditions. We are aware of the idealized conditions. The first step was to check if it is possible to recognize drones and worker bees based on audio signals only, which we have proven to be possible. The next step will be confronting the proposed methods with data collected in constant way on the beehive side with the LTE module. The ground truth will be achieved by visual confirmation of honeybee type on video recordings, as achieved before. For this real-life application of the theoretical solution, we need a data acquisition and wireless communication module, which will be able to transfer the data over the cellular network and send specific alarms defined by a beekeeper. The system should be able to send an update on the status of honeybee colony at given time or on demand. The proposed system can be enlarged by adding more sensors and extending the daily reports with additional data about, for example, weather conditions outside of a beehive and temperature inside a beehive.

Currently, we are developing a wireless communication system for audio data collection. Our hardware includes a radio modulus and a digital signal processor (DSP). The DSP has hardware support to perform the convolution operations used for example in the MUSIC and autoencoder algorithms. The preprocessing can be computationally quite costly. However, neural networks, aside from the training phase, can be highly efficient and fast in terms of computation. The computational complexity of the MUSIC preprocessing method depends on the number of operations: for the estimation of M×M autocorrelation matrix for signal frames of length *N* samples (equal to $N\cdot M^{2}$, where M<<N) and for eigenvalue decomposition of complexity $O(M^{3})$. For example, for the case of NN2 with two hidden layers in the encoder, the computational complexity of the already trained autoencoder neural network is $O(n_{0}\times n_{1}+n_{1}\times n_{2}+n_{2}\times n_{3}+n_{3}\times n_{4}+n_{4}\times n_{5})$, where $n_{i}$ is a number of neurons in the *i*th layer, i=0,1,…,5. In our test, the number of neurons in the input layer is $n_{0}=512$, in the coder layer, it is $n_{3}=8$, and in the output layer, $n_{5}=512$.

The proposed wireless system will not acquire data continuously, as a real-time system is not necessary. Instead, we plan to collect the audio data in short intervals (e.g., 1 s) followed by a 2 s break for processing. The numbers of worker bees and drones bees will be stored in two counters and when the ratio of drones to worker bees exceeds a chosen value, an alarm will be sent to the beekeeper via the GSM network. Further testing for the MUSIC preprocessing method is necessary to accurately define the system parameters and ensure its reliability.

## 6. Conclusions

In this article, we investigated and proposed an early detection method for swarming by determining the most effective preprocessing method for audio signals. We have compared the results for four different methods for signal representation in frequency domain, including mel-frequency cepstral coefficients, gammatone cepstral coefficients, and the MUSIC and Burg algorithms. The extracted features were applied to an autoencoder neural network. To separate the classes of worker bees and drones, the maximum likelihood threshold T* was adopted in the classification part of data processing.

The highest variability of the results can be seen for the standard threshold T1, for which the best performance was obtained for MUSIC feature extraction, followed by the Burg method in second place. Both spectral methods were significantly more accurate for all autoencoder structures compared to the cepstral methods, GTCC and MFCC, for which the results were several percentage points lower. For the 2-sigma threshold T2, and the 3-sigma T3, we observe much smaller variability in the results between all four feature extraction methods, mainly varying in the range 96.87–99.86%.

Even lower accuracy variability between 99.66 and 99.97% is obtained for the optimal threshold T*. This was possible only due to the high separability of the probability distributions of autoencoder reconstruction errors in classes of worker bees and drone bees. In that case, the best classification result was again achieved by the MUSIC spectral method.

The final conclusion is that the MUSIC feature extraction method performed the best for all proposed threshold values T1, T2, T3, and optimal T*, achieving the highest accuracy and the highest F1-score. The spectral methods, Burg and MUSIC, generally use higher-dimensional signal representations, and the insufficient number of hidden layers of the encoder (as for the NN1 case with only one hidden layer) negatively affects the results in comparison to more extensive autoencoder structures. With that constraint, the spectral methods Burg and MUSIC outperformed the cepstral methods GTCC and MFCC for the standard value of threshold T1, commonly used in anomaly detection problems.

Spectral methods, particularly parametric approaches such as the MUSIC algorithm and Burg’s spectral estimation, offer significantly higher resolution in the frequency-domain representation of a signal. It is therefore unsurprising that these spectral methods outperform cepstral methods in our application. Cepstral techniques, like mel-frequency cepstral coefficients (MFCCs), were originally designed for specific signal types, such as voice parameterization, and are commonly employed as default methods for audio signal processing. However, our experimental results demonstrate that for non-standard signals, such as insect buzzing, cepstral methods yield lower performance compared to spectral methods.

The proposed solution for audio classification of honeybees can be further developed for drone bee detection, facilitating the creation of an intelligent counting system for beekeepers. Future research should experimentally assess whether an autoencoder trained on data from one beehive can generalize effectively to another beehive and evaluate the impact of environmental noise in new settings on the accuracy of honeybee classification.

Data collection for this study was conducted over a brief period on a clear, sunny day, resulting in a database of clean recordings. Given the anomaly detection capability of autoencoder neural networks, it is likely that environmental noise or rain would be erroneously classified as anomalies, akin to the drone bee class. In future work, we intend to address the challenge of noisy data by collecting a more comprehensive dataset. While the classification method presented in this manuscript may currently yield low accuracy in outdoor scenarios, it provides valuable insights into which frequency-domain signal representation methods most effectively capture the distinguishing features between the two classes. 

## Figures and Tables

**Table 1:** Classification accuracy for class separation by thresholds T1, T2, T3, and T*.

Feature	Autoencoder	Accuracy	Accuracy	Accuracy	Accuracy
Extraction	Structure	for T1	for T2	for T3	for T*
**Burg**	NN1	95.72%	98.03%	98.60%	98.90%
	NN2	96.57%	98.85%	99.58%	99.61%
	NN3	96.43%	98.84%	99.60%	99.61%
	NN4	96.40%	98.86%	99.65%	99.66%
**GTCC**	NN1	92.33%	97.62%	98.87%	99.93%
	NN2	92.06%	97.69%	98.94%	99.93%
	NN3	91.64%	97.91%	98.98%	99.94%
	NN4	92.48%	97.82%	98.89%	99.92%
**MFCC**	NN1	93.89%	97.65%	98.91%	99.89%
	NN2	93.80%	97.65%	98.99%	99.82%
	NN3	93.91%	97.68%	98.93%	99.86%
	NN4	93.78%	97.82%	98.95%	99.85%
**MUSIC**	NN1	96.87%	96.87%	98.54%	99.55%
	NN2	**99.59%**	**99.85%**	**99.86%**	99.89%
	NN3	97.69%	98.94%	99.54%	**99.97%**
	NN4	93.99%	96.89%	98.79%	99.89%

The highest accuracy in bold font.

## Data Availability

Data are available in a publicly accessible repository. The data presented in this study are openly available in “Dataset for honey bee audio detection” at https://zenodo.org/doi/10.5281/zenodo.10359685 (accessed on 9 July 2024), reference number [37].

## Abbreviations

The following abbreviations are used in this manuscript:

AR	Autoregressive process
ARMA	Autoregressive moving average process
CNN	Convolutional neural networks
DCT	Discrete cosine transform
FFT	Fast Fourier transform
GTCC	Gammatone cepstral coefficients
LSTM	Long short-term memory
MA	Moving average process
MFCC	Mel-frequency cepstrum coefficient
MLP	Multilayer perceptron
MSE	Mean square error
MUSIC	Multiple signal classification
PSD	Power spectral density
RNN	Recurrent neural network
STFT	Short-time Fourier transform
SVM	Support vector machine
